# A case of toxoplasmic encephalitis in a patient on cancer chemotherapy in Uganda

**DOI:** 10.4314/ahs.v24i4.14

**Published:** 2024-12

**Authors:** Yekosani Mitala, Raymond Atwine, Abraham Birungi, Ambaru Jacinta, Kuraishi Baluku, Semei Sekitene, Edwin Nuwagira

**Affiliations:** 1 Department of Pathology, Mbarara University of Science and Technology, Mbarara city, Uganda; 2 Uganda Cancer Institute-Mbarara Satellite site, Mbarara city, Uganda; 3 Department of Medicine, Mbarara University of Science and Technology, Mbarara city, Uganda; 4 Tuberculosis treatment unit, Mbarara Regional Referral Hospital, Mbarara city, Uganda

**Keywords:** Toxoplasmosis, Uganda, sub-Saharan Africa, case report

## Abstract

**Background:**

Reactivation of central nervous system (CNS) toxoplasmosis can be caused by immunosuppression (ISS) of any kind. However, anti-cancer chemotherapy combined with human immunodeficiency virus (HIV) induced ISS results in an atypical presentation that is fatal.

**Case presentation:**

A 46 years old man with a well-controlled infection of the human immunodeficiency virus presented with generalized tonic-clonic seizures following the second dose of anti-cancer chemotherapy for esophageal cancer. His brain's computerized tomography (CT) scan showed enlarged ventricles with no space-occupying lesions. Cerebrospinal fluid (CSF) smears stained with hematoxylin and eosin (H&E) revealed numerous bradyzoites and tachyzoites consistent with central nervous system toxoplasmosis.

**Conclusion:**

With a double burden of cancer and Human immunodeficiency virus (HIV) infection in low-income countries, this case raises awareness about the atypical presentation of CNS toxoplasmosis reactivation among patients on cancer chemotherapy.

## Introduction

Immunosuppression is associated with the reactivation of several latent infections and the acquisition of opportunistic diseases^1^. CNS toxoplasmosis in particular is uncommon and rarely causes serious disease in immune-competent people. In immunocompromised people like cancer patients on chemotherapy and HIV patients, toxoplasmosis is fatal^2^. Toxoplasmosis has been fairly studied among HIV and cancer patients but information about its prevalence in cancer patients co-infected with HIV is scarce. Available lierature suggests that HIV patients with cancer on chemotherapy have higher incidence rates of opportunistic infections than those with HIV and no cancer^3^. Toxoplasmosis is caused by the tissue protozoa; Toxoplasma gondii, a ubiquitous intracellular organism that has a predilection for the CNS. The infection is acquired through the ingestion of undercooked meat contaminated with tissue cysts or oocysts from cats' feces^4^. It is usually a reactivation of latent infection and is associated with late-stage HIV among patients with CD4+ T-cell counts below 200 cells/µl although it has also been reported among patients on anti-cancer chemotherapy^5^. The active form of the disease is uniformly fatal especially in the immunocompromised hosts if not properly treated^6^. CNS toxoplasmosis causes focal or multiple necrotizing ring-enhancing lesions with edema and mass effect in the deep into the white matter^7^. Occasionally, it causes diffuse non-necrotic encephalitis with no obvious characteristic ring-enhancing lesions. The latter is more frequently associated with HIV induced immunosuppression with very few case of the same so far reported in cancer patient^8^,^9^. In this case, we report a case of diffuse non-necrotizing encephalitic toxoplasmosis in a person with well controlled HIV and esophageal cancer on chemotherapy at a tertiary hospital in Uganda.

## Case description

A 46 years old man who had been living with HIV on combination antiretroviral therapy (cART) of Tenofovir disoproxil Fumarate (TDF), Lamivudine (3TC), and Dolutegravir (DTG), for three years. He had been previously diagnosed with esophageal adenocarcinoma (stage III) for which he was receiving chemotherapy (Epirubicin and Cisplatin). His CD4+ T cell count prior to the cancer diagnosis was 754 cells/µl with undetectable HIV RNA (viral load). He presented with headache, photophobia, fever, and vomiting that had been on for a week prior to this admission. One day before admission, he developed convulsions that were not relieved by any of the available antiepileptic drugs (AEDs).

## Clinical examination findings

On physical examination, we found a well-nourished man. He did not have any pale or jaundiced mucous membranes, nor any palpable lymph nodes. He had neck stiffness, a negative kerning's sign, and dilated pupils about 6 mm all equal but sluggishly reacting to light. There was no obvious facial asymmetry or focal neurology. The abdomen was mildly distended, with tenderness in the epigastrium, a positive fluid thrill, and no palpable masses or enlarged organs. The cardiovascular and respiratory exams were normal.

## Timeline

Patient had been in cART for 3 years prior to the cancer diagnosis, received 2 cycles of chemotherapy with episodes of neutropenia in between. Five days after the second cycle, he was readmitted with signs of meningitis and had lost consciousness. A diagnosis of CNS toxoplasmosis was reached at and treatment started. Seeing no significant improvement, the care giver requested to allow them home from where the patient died 3 days later.

## Diagnostic assessment

A complete blood count, liver enzymes, urea, creatinine, hepatitis Band C, and Malaria were all unremarkable. His chest CT scan was also normal. On suspicion of brain metastases, a head CT scan was done (Figure 1). His family consented for a lumbar puncture which was done and 30 ml of CSF was drained. Analysis of hematoxylin and eosin (H&E) stained CSF smears showed presence of Toxoplasma gondii of various forms. Based on the clinical presentation and findings of head CT scan, H&E stained CSF smear, and CSF chemistry, a diagnosis of diffuse non-necrotic cerebral toxoplasmosis was made. Differential diagnoses included; bacterial meningitis, cryptococcal meningitis, metastasis, disseminated tuberculosis, malaria etc were all excluded clinically. Other details of the lumbar puncture, CSF analysis, and other laboratory values are described in Table 1.

**Appendix I:** CSF analysis findings

CSF analysis	Macroscopic examination: Opening pressure >40 cm H_2_O Colorless. **Cell counts:** White cell count 0.7x10^6^ cells/l (0-5) Differentials: ⚬ Neutrophils 49%, ⚬ Lymphocytes 32%, monocytes 19%. Indian Ink: Negative Gram stain: No organisms seen Zeihl-Neelsen stain: No Acid Fast Bacilli seen Biochemical tests: Protein 0.3g/l (0.15-0.40 g/l), Glucose 5.5 mmol/l (2.5-4.0 mmol/l)
CSF sediment analysis	Hematoxylin and Eosin: Numerous cysts with bradyzoites and free tachyzoites. **(Figure 2)**

## Treatment

The patient was started on treatment for CNS toxoplasmosis with (pyrimethamine 200mg, followed by 50mg daily, and Clindamycin tablets 300mg daily by a nasogastric tube to complete six weeks). He also received Folic acid and intravenous dexamethasone 8 mg daily. Five days later, the frequency of convulsions had reduced but the patient was still aphasic and confused.

## Follow-up and outcome

When the care giver saw dismal improvement after 5 days of hospitalization coupled with the high in patient expenses, he requested for discharge. They received professional counselling and then discharged as requested. They were contacted after 4 days and we discovered that the patient had died a day earlier

## Discussion

Our case represents the first example of diffuse non-necrotic encephalitis caused by toxoplasmosis in a profoundly immunosuppressed patient with HIV and Cancer diagnosed antemortem. Such atypical presentations can pause diagnostic challenges especially in settings with limited diagnostic capacity like ours. Typically, patients with diffuse encephalitic toxoplasmosis present with seizures, confusion, and altered mental state with rapid progression to death as seen in our case. On the contrary, the necrotic type of toxoplasmosis causes abscess formation, and focal neurological signs like hemiparesis, dysphasia, etc predominate. However, the mass effect may as well cause seizures as seen in the diffuse type^10^. Notably, brain CT scan in toxoplasmic encephalitis are normal as also evident in our case (see fig 1). Occasionally, repeat CT scans done on a later date may reveal ring-enhancing lesions as seen in one case of heart transplant recipient^11^. Magnetic resonance imaging (MRI) has better sensitivity compared to CT scan and can detect lesions not seen on CT scan^7^, however, MRI is not available at our hospital and so it was not done. The characteristic ring-enhancing lesions of focal or multifocal necrotic toxoplasmosis are not usually seen. CSF analysis is of debatable utility. CSF chemistry is usually not conclusive although some authors mention CSF leukocytosis as the best predictor of infection but this is often not seen. In our case, CSFchemistry was largely normal (see table 1) similar to findings reported by several authors^7^. Astonishingly, microscopic examination of H&E stained CSF centrifuged sediments revealed presence of bradyzoites of toxoplasma and free tachyzoites (see fig 2). Bradyzoites are a life stage of toxoplasma gondii found in tissues and replicate slowly while the tachyzoites are at the rapidly growing life stage. The bradyzoites containing hundreds of parasites are surrounded by a clear space that corresponds to a thin capsule that does not stain with H&E. The capsule is also Periodic acid Schiff (PAS) negative. H&E examination of CSF is not routinely done in suspected toxoplasmosis. The only available literature suggests use of Giemsa stain on CSF centrifuged sediments^9^. Most studies have used CSF or blood to detect toxoplasma antigens, antibodies or nucleic acids using polymerase chain reaction (PCR), and several other methods^12^-^14^. However, because our patient was severely immunosuppressed, we bet he could raise detectable amounts of antibodies. Also, serology for these antibodies is also not available in our hospital.

Research has shown that the risk of opportunistic infections in virally suppressed HIV patients with a CD4 count of more than 200 cells/µl is almost similar to immunocompetent persons^3^. In our patient, the CD4 cell count prior to cancer diagnosis was 754 cell/µl and he had an undetectable viral load. However, because esophageal cancer causes difficulty in swallowing, we suspect that his level of compliance to cART was low at the time. This might have caused a rise in the viral load, plummeting the CD4 cell count. Coupled with the effects of chemotherapy on the CD4 cells, we think this caused profound immunosuppression that resulted in the reactivation of the latent toxoplasma infection resulting in diffuse non-necrotic toxoplasmic encephalitis. In less immunosuppressed individuals, necrotic focal or multifocal ring-enhancing lesions are caused by the body's immune system fighting the infection resulting in tissue death (necrosis). This implies that our patient was profoundly immunosuppressed to mount a significant response against the infection.

Our patient was treated with a combined therapy of pyrimethamine, clindamycin, folic acid and intravenous dexamethasone to reduce the brain swelling. Treatment was intended to last for 6 weeks, but due to lack of cooperation by the care givers this was not possible and so the patient died after being prematurely discharged.

## Limitations and challenges

We had several challenges when managing this case. First, we were unable to do serial CD4 cell count during chemotherapy and so we were unable to determine the extent to which it lowered the CD4 cell count. The limited investigative capacity limited our list of differential diagnoses. MRI is superior to CT scan in detecting brain lesions in patients with CNS toxoplasmosis, its absence impacted our level of certainty of the diagnosis. Also, the lack of patience by the patients, care giver limited our follow-up time and because the patient died from home, postmortem was not done to further determine the existent of damage that the disease had caused.

## Ethical concern and consent

We sought assent from the next of kin so that we could perform all the medical interventions as well as to have this case published. We also had an ethical dilemma of allowing the patient be taken home although he was not yet stable which might have partly costed his life.

## Conclusion

As the number of people living with HIV and cancer increases, this case raises awareness about the risk of reactivation of CNS toxoplasmosis and may not present with characteristic ring-enhancing lesions. There is a need to conduct larger studies to determine the prevalence of toxoplasmosis infection among persons living with HIV and cancer.

## Patient perspective

We were only able to obtain the caretakers perspective since the patient was not fully conscious. Right from the beginning, the care giver had no hopes of recovery for his brother because of the multimorbidities he had. This was further complicated by the poor economic status of the family since most of the drugs were not readily available in the hospital and they had to be bought from private pharmacies outside the hospital. Not surprising, he had to request for discharge prematurely.
